# Progress Toward Poliomyelitis Eradication — Pakistan, January 2019−September 2020

**DOI:** 10.15585/mmwr.mm6946a5

**Published:** 2020-11-20

**Authors:** Christopher H. Hsu, Muhammad Shafiq-ur Rehman, Kelley Bullard, Jaume Jorba, Milhia Kader, Hamish Young, Muhammad Safdar, Hamid S. Jafari, Derek Ehrhardt

**Affiliations:** ^1^Global Immunization Division, Center for Global Health, CDC; ^2^World Health Organization, Islamabad, Pakistan; ^3^IHRC, Inc., Atlanta, Georgia; ^4^Division of Viral Diseases, National Center for Immunization and Respiratory Diseases, CDC; ^5^United Nations Children's Fund, Islamabad, Pakistan; ^6^National Emergency Operation Center, Islamabad, Pakistan; ^7^World Health Organization, Amman, Jordan.

Pakistan and Afghanistan are the only countries where wild poliovirus type 1 (WPV1) is endemic (*1*,*2*). In 2019, Pakistan reported 147 WPV1 cases, approximately 12 times the number reported in 2018. As of September 15, 72 cases had been reported in 2020. Since 2019, WPV1 transmission has also spread from Pakistan’s core poliovirus reservoirs (Karachi, Peshawar, and Quetta block) to southern districts of Khyber Pakhtunkhwa (KP), Punjab, and Sindh provinces. Further, an outbreak of circulating vaccine-derived poliovirus type 2 (cVDPV2), first detected in July 2019, has caused 22 paralytic cases in 2019 and 59 as of September 15, 2020, throughout the country. The coronavirus disease 2019 (COVID-19) pandemic has substantially reduced delivery of polio vaccines through essential immunization (formerly routine immunization) and prevented implementation of polio supplementary immunization activities (SIAs)* during March–July 2020. This report describes Pakistan’s progress in polio eradication during January 2019−September 2020 and updates previous reports (*1*,*3*,*4*). The Pakistan polio program has reinitiated SIAs and will need large, intensive, high-quality campaigns with strategic use of available oral poliovirus vaccines (OPVs)^†^ to control the surge and widespread transmission of WPV1 and cVDPV2.

## Immunization Activities

**Essential immunization.** Based on a national survey of 12,815 households in 2017–2018, essential immunization coverage with 3 doses of bivalent OPV (bOPV, containing vaccine virus types 1 and 3) by age 1 year was 86% and with 1 dose of inactivated poliovirus vaccine (IPV), which contains all three serotypes, was 64% (*5*). Coverage in 2019 was highest in Azad Jammu and Kashmir (92%) and Islamabad (95%) and lowest in Balochistan (66%) and Gilgit-Baltistan (67%). Provincial essential immunization coverage with bacillus Calmette–Guérin, OPV3, pentavalent (diphtheria, tetanus, pertussis, hepatitis B, and *Haemophilus influenzae* type b), and measles vaccines decreased 22%–49% from 2019 to 2020 (*6*).

Vaccination histories of children aged 6–23 months with acute flaccid paralysis (AFP) who tested negative for wild poliovirus and vaccine-derived poliovirus (VDPV) (nonpolio AFP [NPAFP]^§^) are a surrogate estimate of population polio vaccination coverage. In 2019, the highest proportion of children with NPAFP who had not received any polio vaccination (zero-dose children) were in Balochistan (3.9%) and KP (1.1%).

**Supplementary immunization activities.** In 2019, three national and four subnational SIAs were conducted using bOPV. Because of COVID-19 outbreaks during January–September 2020, only two national campaigns (February and September using bOPV) and two subnational campaigns (March and August) using monovalent OPV type 2 (mOPV2) were conducted. In addition, multiple small-scale vaccination campaigns were implemented in response to isolation of poliovirus from environmental surveillance (sewage sampling) or persons with AFP, using bOPV for WPV1 or mOPV2 for cVDPV2. SIA quality was assessed through intracampaign monitoring surveys and lot quality assurance sample (LQAS) surveys.^¶^ During the national immunization days (NIDs) conducted in January 2019 (i.e., before the COVID-19 pandemic), one province (Gilgit-Baltistan) did not meet LQAS targets (≥80%), compared with three (Azad Jammu and Kashmir, Gilgit-Baltistan, and Sindh) during the September 2020 NID (i.e., during the COVID-19 pandemic). 

**Community-based vaccination.** Since 2015, locally recruited community-based vaccinators have engaged with communities in core poliovirus reservoirs to increase vaccination during and between SIAs. Community-based vaccinators, recruited from the local population, are mostly (85%) female, which facilitates entry in homes in religiously conservative areas, and are perceived as possessing a vested interest in reaching all children in their communities. As of August 2020, a total of 10,318 community-based vaccinators, approximately one half the community-based vaccinator workforce of August 2019, have been deployed in areas at high risk.

## Surveillance Activities

**AFP surveillance.** In 2019 and 2020, all provinces exceeded the target NPAFP case rate of six per 100,000 persons aged <15 years and the 80% target proportion of AFP cases with collection of adequate specimens** (Table). During January−September 2020, the national NPAFP rate was 14.2 (range = 10.2–18.9 among provinces), lower than that during the same period in 2019 (20.5; range = 16.3–31.0) (*1*). The national percentages of AFP cases with adequate stool specimens were 88% in 2019 and 89% in 2020.

**TABLE:** Acute flaccid paralysis (AFP) surveillance indicators, number of reported cases of wild poliovirus (WPV), and number of reported cases of circulating vaccine derived poliovirus type 2 (cVDPV2), by region and period — Pakistan, January 2019-September 2020

Indicator	Pakistan Total	Region						
		Azad Jammu Kashmir	Gilgit-Baltistan	Islamabad	Khyber Pakhtunkhwa	Punjab	Balochistan	Sindh
**AFP surveillance indicators (2019–2020)**								
No. of AFP cases (2019)	**15,216**	388	156	188	3,366	7,287	658	3,173
No. of AFP cases (2020)	**7,698**	147	78	80	1,797	3,609	352	1,635
Nonpolio AFP rate (2019)*	**20.5**	18.2	22.4	31.0	21.8	16.9	16.3	16.9
Nonpolio AFP rate (2020)*	**14.2**	10.2	16.5	18.9	17.2	12.0	12.4	12.0
% with adequate specimens (2019)^†^	**88**	91	86	87	83	87	89	90
% with adequate specimens (2020)^†^	**89**	90	88	87	85	89	91	91
**Reported WPV cases**								
Jan–Jun 2019	**44**	0	0	0	34	5	2	3
Jul–Dec 2019	**103**	0	0	0	59	7	10	27
Jan–Sep 2020	**72**	0	0	0	22	10	18	22
**Total**	**219**	0	0	0	115	22	30	52
**Reported cVDPV2 cases**								
Jul–Dec 2019	**22**	0	4	1	16	1	0	0
Jan–Sep 2020	**59**	0	0	0	42	11	1	5
**Total**	**81**	**0**	**4**	**1**	**58**	**12**	**1**	**5**

* Per 100,000 children aged <15 years.

^†^ Two stool specimens collected at an interval of at least 24 hours within 14 days of paralysis onset and arriving in a World Health Organization–accredited laboratory with reverse cold chain maintained and without leakage or desiccation.

**Environmental surveillance.** AFP surveillance is supplemented through systematic sewage sampling (currently at 60 regular sampling sites and eight ad hoc sites) and testing for poliovirus. During January 2019–September 2020, WPV1 has been detected in all provinces, including core reservoirs (Karachi, Peshawar, and Quetta block) and multiple sites in Balochistan, KP, Punjab, and central and northern Sindh. Among the 68 sampling sites, the proportion of positive samples detected in 2020 (55%) increased compared with that during the same period in 2019 (43%). VDPV2 was first detected through environmental surveillance in August 2019 in Gilgit-Baltistan and northern KP. Over the next 12 months VDPV2 was detected in Balochistan, Punjab, and Sindh; by February 2020, VDPV2 was detected at 12 (18%) of the 68 sampling sites in all provinces.

**FIGURE 1:**
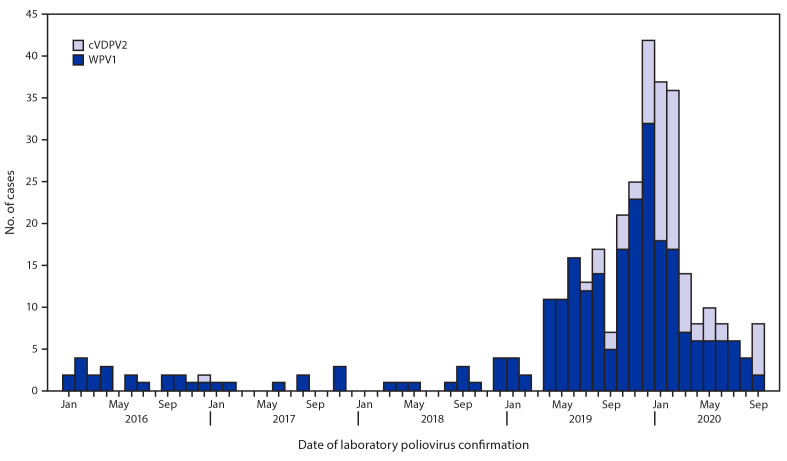
Wild poliovirus type 1 (WPV1) and circulating vaccine-derived poliovirus type 2 (cVDPV2) cases, by month — Pakistan, January 2016–September 2020

**FIGURE 2:**
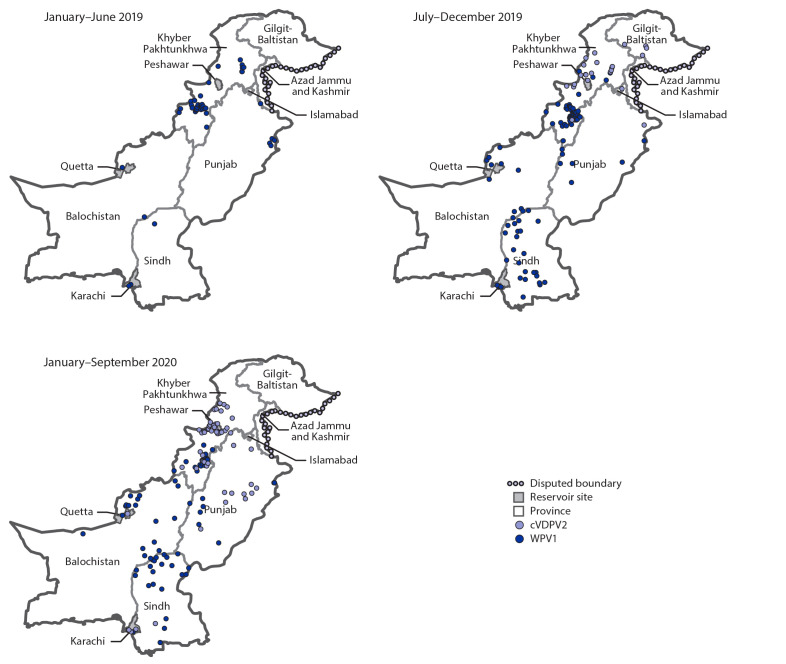
Location of cases of wild poliovirus type 1 (WPV1) and circulating vaccine-derived poliovirus type 2 (cVDPV2), by province and period — Pakistan, January 2019–September 2020

**Epidemiology of WPV1 and cVDPV2 cases.** During 2019, 147 WPV1 cases were reported in Pakistan, more than 12 times the 12 reported cases during 2018 (Figure 1). During January–September 2020, 72 WPV1 cases had been reported among 33 districts in four provinces, compared with 72 from 22 districts in four provinces during the same period in 2019. Among the 219 WPV1 cases with paralysis onset during January 2019−September 2020, 115 (53%) were from KP, 30 (14%) from Balochistan, 52 (24%) from Sindh, and 22 (10%) from Punjab (Table) (Figure 2). Ages of the 219 WPV1 patients ranged from 1 to 168 months (median 18 months). Thirty-three (15%) patients were zero-dose children, and 153 (70%) had received ≥4 doses.

Several WPV1 genetic lineages persisted through the 2019–20 low season (November–April). Among the 10 genetic clusters (groups of polioviruses sharing ≥95% sequence identity in the viral capsid protein [VP1]) associated with AFP cases during the reporting period, seven were detected during the low season, mostly in KP and Sindh provinces.

Since the detection in July 2019 of the first cVDPV2 case in Diamer district (Gilgit-Baltistan province) through September 15, 2020, a total of 81 cases affecting 30 districts in six provinces had been reported (Table) (Figure 1). The 2019 cVDPV2 outbreaks occurred as five independent emergences (*7*); in 2020, the cVDPV2 cases were genetically linked to two of the five original cVDPV2 emergences. Fifty-eight (72%) cases were from KP, one (1%) from Balochistan, five (6%) from Sindh, 13 (16%) from Punjab, and four (5%) from Gilgit-Baltistan. Ages of cVDPV2 patients ranged from 6 to 102 months (median 16 months). Ten (12%) were zero-dose children, 41 (51%) had never received essential immunization, 13 (16%) had received mOPV2, and 17 (21%) had received IPV. Three breakthrough cVDPV2 transmissions have been detected after response vaccination campaigns: one each in Nowshera and Torghar districts in KP and Rawalpindi district in Punjab; all occurred before March 2020.

## Discussion

Significant setbacks in Pakistan’s polio eradication began during 2018, with a sharp increase in WPV1 cases and positive environmental samples for WPV1 and cVDPV2 in 2019. The WPV1 resurgence resulted from ongoing challenges reaching children in districts with endemic transmission and deterioration in overall SIA quality. Efforts to halt transmission of WPV1 failed in 2019, and with emergence of cVDPV2, spread of both poliovirus types was exacerbated in 2020 by the COVID-19 pandemic, which placed significant demands on the health care system and disrupted surveillance and vaccination activities. The COVID-19 response required leadership, laboratory personnel, and frontline workers from polio surveillance and vaccination activities to combat rising COVID-19 cases, resulting in reduction of essential immunization and suspension by the National Emergency Operations Center of all polio SIAs during March–July 2020.

SIAs need to be implemented with great urgency to close immunity gaps created during the first half of 2020. The full range of polio vaccines available (mOPV2, bOPV, and trivalent OPV [tOPV, containing vaccine types 1, 2 and 3]) can be strategically distributed to reduce transmission, depending on the type-specific serotype circulating and population immunity. In June 2020, the Pakistan Technical Advisory Group^††^ recommended using tOPV in SIAs, to immunize against types 1 and 2 simultaneously. The National Emergency Operations Center resumed SIAs with a small-scale mOPV2 campaign in July, followed by a larger-scale mOPV2 campaign in August and a bOPV NID in September. NIDs are planned for every 5 weeks from September to December using tOPV, if available, or bOPV.

Pakistan’s polio program would also benefit from gaining community trust through effective messaging to counter false information and vaccine refusals. Vulnerable, high-risk areas include Quetta, Sindh province, districts of Khyber and Peshawar, and southern districts of KP province. Efforts to improve community engagement and relevant social data collection are necessary to understand challenges hindering vaccine acceptance in these high-risk areas. Community-based vaccinators have improved vaccination coverage by improving trust in some communities at high risk, and by downsizing its workforce in 2020 to place the most skilled community-based vaccinators where they might have the greatest impacts. Community-based vaccinators will continue to play a crucial role in vaccinating hard-to-reach children.

Mistrust of vaccines and vaccinators is also responsible for poor vaccination coverage among certain migrant and displaced communities. Some of these groups move back and forth between Pakistan and Afghanistan or settle along the border in KP and Balochistan. Culturally sensitive efforts with direct engagement of these communities, including adequate representation within the local vaccination workforce, will be needed to improve vaccine acceptance. In addition, coordination between Pakistan and Afghanistan through data sharing, SIA coordination, and cooperative border health efforts, will be essential to eradicating WPV1 from the border (*2*).

The national program and the Global Polio Eradication Initiative^§§^ partners in Pakistan have undergone significant transformation since late 2019 to overcome challenges in oversight, data burden, and efficiency. Transformation goals included improving accountability of field activities; restructuring and defining government and partnership roles; and streamlining data flow from the union council and district levels to provincial and national levels. The new program structure needs to be finalized and commence functioning immediately to reverse the trend of nationwide WPV1 and cVDPV2 expansion and ultimately to achieve regional and global polio eradication.

**Summary**

**What is already known about this topic?**

Since 2016, Pakistan and Afghanistan are the only countries to report ongoing transmission of indigenous wild poliovirus type 1 (WPV1) and remain the last countries where polio is endemic.

**What is added by this report?**

WPV1 transmission continued in Pakistan during January 2019–September 2020, and circulating vaccine-derived poliovirus type 2 (cVDPV2) outbreak cases have spread throughout the country since July 2019. In 2020, the coronavirus disease 2019 pandemic has substantially reduced delivery of polio vaccines.

**What are the implications for public health practice?**

Stopping WPV1 and cVDPV2 transmission in Pakistan will require resumption of high-quality national vaccination campaigns with strategic use of oral poliovirus vaccines, continuing cross-border coordination with Afghanistan, national coordination among the partnerships, and gaining the trust of high-risk communities.

## Footnotes

* Mass campaigns conducted for a brief period (days to weeks) in which 1 dose of oral poliovirus vaccine is administered to all children aged <5 years, regardless of vaccination history. Campaigns can be conducted nationally or subnationally.

† Three types of oral poliovirus vaccine are currently in use in Pakistan: monovalent OPV to type 2 (mOPV2), bivalent OPV (bOPV) to types 1 and 3, and trivalent OPV (tOPV) to types 1, 2, and 3.

§ Vaccination histories of children aged 6–23 months with acute flaccid paralysis who do not receive positive test results for WPV/VDPV are used to estimate OPV coverage of the overall target population and to corroborate national reported routine vaccination coverage estimates.

¶ Lot quality assurance sample survey is a two-stage post-campaign assessment: stratified random sampling and one-sided hypothesis testing during analysis. Lots are union councils (the tier of government below districts) within a vaccination catchment area. If the number of vaccinated children identified by finger marking within a lot has met the predetermined threshold, the lot has passed.

** AFP surveillance quality is monitored by performance indicators that include 1) the detection rate of nonpolio acute flaccid paralysis (NPAFP) cases and 2) the percentage of AFP cases with adequate stool specimens. World Health Organization (WHO) operational targets for countries with endemic-poliovirus transmission are NPAFP detection rates of two or more cases per 100,000 population aged <15 years and adequate stool specimen collected from ≥80% of AFP cases. For Pakistan, an endemic country, the minimum detection rate is six per 100,000 population aged <15 years. Adequate stool specimen is defined as two stool specimens collected ≥24 hours apart, both within 14 days of paralysis onset, and shipped on ice or frozen packs to a WHO-accredited laboratory, arriving in good condition (i.e., without leaks or desiccation) within 3 days.

†† The Technical Advisory Group includes international polio experts who meet biannually to assess Pakistan’s eradication activities and progress and to provide recommendations toward the goal of eradication.

§§ The Global Polio Eradication Initiative is a partnership of CDC, the Bill and Melinda Gates Foundation, WHO, United Nations Children's Fund, and Rotary International.
